# Stinging Nettle (*Urtica dioica*) Roots: The Power Underground—A Review

**DOI:** 10.3390/plants14020279

**Published:** 2025-01-19

**Authors:** Françoise Martz, Santeri Kankaanpää

**Affiliations:** 1 Production System Unit, Natural Resources Institute Finland, 96200 Rovaniemi, Finland; 2 Production System Unit, Natural Resources Institute Finland, 31600 Jokioinen, Finland; santeri.kankaanpaa@luke.fi

**Keywords:** lectin, agglutinin, rhizome, sitosterol, lignans, scopoletin, antifungal, antiviral, alternative crop

## Abstract

Stinging nettle (*Urtica dioica*) is an herbaceous perennial plant native to Eurasia, wildly distributed throughout the temperate parts of the world. Although generally considered as a weed due to its fast growth and invasive capacity, stinging nettle is well suited to cultivation and is currently experiencing a revival as a beneficial crop due to its numerous potential applications. This interest reflects in an increasing number of scientific articles related to nettle in the last years. However, reports mostly focus on the aerial parts of the plant. Roots are rich in numerous phytochemicals such as phytosterols, lignans, coumarins, sugars, and lectins. By compiling the most relevant publications, the aim of this review is to gather the current knowledge about nettle roots, such as root system functioning, biochemical composition, and related functional activities. A special emphasis is placed on lectins (or UDA for *Urtica dioica* agglutinin) due to their functional activities. This review highlights the potential of nettle root as a source of biomolecules. Gaps of knowledge and possible future directions for nettle root research, production, and uses are discussed.

## 1. Introduction

Stinging nettle (*Urtica dioica*) is an herbaceous perennial plant that grows wildly throughout the temperate parts of the world. Despite its contemporary reputation as an invading weed, stinging nettle has gained more interest both scientifically and commercially due to its growth properties and multiple applications [1,2,3]. It is currently experiencing a revival as a beneficial crop.

Nettle leaves are rich in antioxidants and health promoting compounds [4,5,6,7], which explains its traditional use as food and medicine. The bast fibers contained in stems have long been used for textiles since ancient times, before being forgotten in the cotton era [1,8]; although it is still suffering from technical bottlenecks, nettle is again attracting high interest as a sustainable source of natural fibers.

Nettle roots, like leaves and stems, are rich in valuable phytochemicals despite having a specific chemical profile [9,10,11]. Particularly, roots are distinguished from aerial parts by their high lectin content, commonly known as *Urtica dioica* agglutinins (UDAs). Several reviews have been published about nettle in general (e.g., [1,8]), or nettle stems for fibers (e.g., [3,12,13]), or leaves for food and medicinal applications [7,10,14,15]. To our knowledge, Chrubasik et al. (2007) [16] is the only review focusing singularly on the components and clinical effectiveness of nettle roots. This lack in root interest likely reflects the fact that, as underground organs, nettle root growth is difficult to monitor, harvesting is laborious, and since stinging nettle is a perennial plant, root harvest is destructive to future growth. However, roots are rich in active substances, and we will try to convince the reader about the strong potential of nettle root as a source of active biomolecules.

The literature cited in this review was primarily collected from the authors’ previous research on nettle [17] and then completed using online search engines, Web of Science (https://www.webofscience.com/, accessed on 7 November 2024), Google Scholar (https://scholar.google.com, accessed on 7 November 2024), and PubMed (https://pubmed.ncbi.nlm.nih.gov/, accessed on 7 November 2024) using appropriate keywords. The publications are rather evenly spread over the years since the 1980s and are increasing with time (Figure S1a), as recently shown [2], with older articles dealing with root functioning and lectin isolation. When looking at the studies by country, ecological and physiological studies from German teams predominated in the 1980s, while more recently, Japanese teams have published several studies related to 3D structure and carbohydrate binding site, and Iranian teams have published several studies related to the health benefits of nettle root phytochemicals (Figure S1b).

## 2. The Nettle Root System

**Figure 1:**
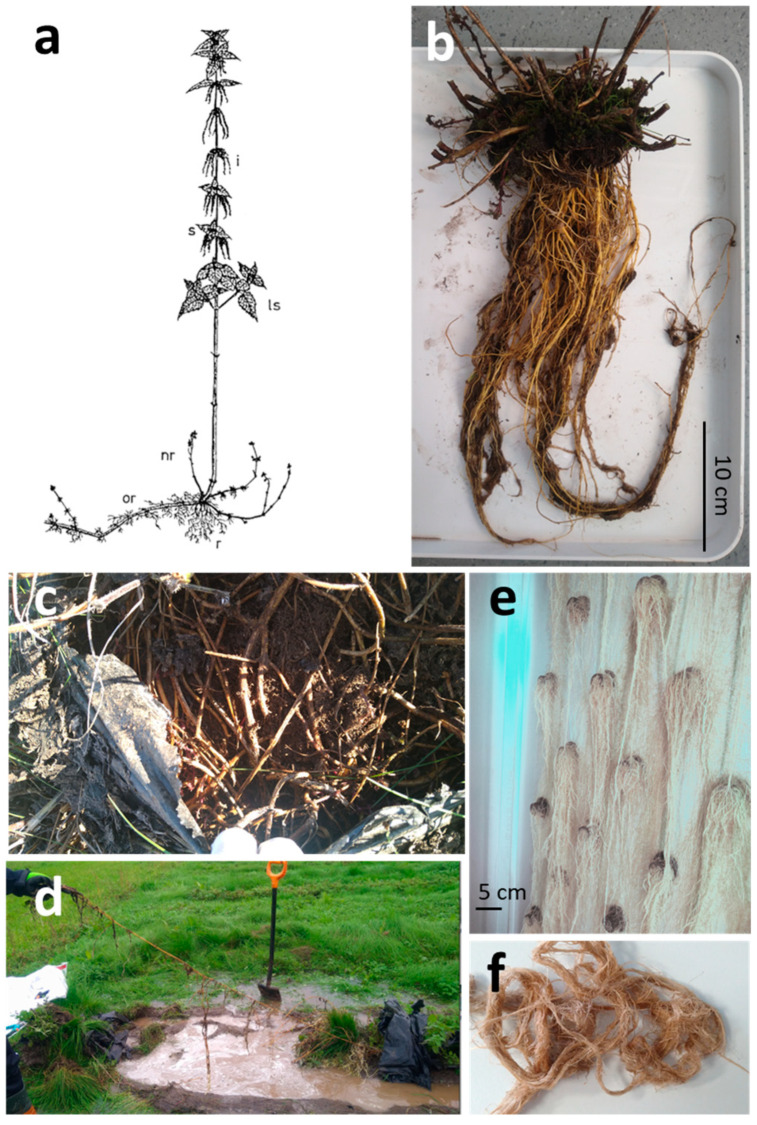
Nettle root system under different cultivation system: (**a**) nettle plant diagram: i, inflorescence; s, stipule; Is, lateral shoot (branch); or, old rhizome; nr, new rhizome; r, roots (from [23]); (**b**) complete root system collected in peat-based soil in Northern Finland, 2 years after planting; (**c**) September roots growing under plastic film mulching 2 years after planting; (**d**) rhizome length 3 years after planting (picture taken after heavy rain); (**e**,**f**) root system in aeroponic cultivation (“360- Low Carbon Investments” project, Lapland University of Applied Sciences, Rovaniemi, Finland, 2023, pictures from S. Sarkkinen). All pictures are from the same accession, originating from Rovaniemi, Finland.

The nettle root system is composed of both roots and rhizomes (Figure 1a). Rhizomes are underground stems, with nodes and internodes and apical and axillary buds that produce the shoot and root systems of new plants. Nettle rhizomes grow horizontally and can form a dense mat in the upper soil layer and have the same structural features as roots [18]. Older rhizomes and roots are both covered with a yellow cork layer [19].

Although tap root is sometimes mentioned and nettle acknowledged for its deep root system, from our experience, tap roots remained difficult to identify and the root system was mainly found in the upper layer of the soil (Figure 1). No tap root is described by Šrůtek et al. [19,20] either. New reddish rhizomes are mainly produced in late summer or autumn, either from older rhizomes or from the stem bases. These rhizomes bear scale leaves with a small rudimentary lamina and large stipules. Roots develop immediately above the stipules (four per node) so numerous laterals rhizomes are rapidly forming and most shoots develop from the new horizontal rhizomes in the autumn [20]. The architecture of the rhizome system may change under conditions of soil water storage and the growth pattern of the new rhizome is affected by soil fertility and competition [19,20]. Seeds are necessary for the colonization of new sites but as soon as the stinging nettle has established a large and dense population [21], longer rhizomes can probably also evade the resource-depletion zone, such as at the edge of large old clones [20] (Figure 1).

The different modes of propagation between *U. dioica* (perennial, mainly rhizomatic propagation) and its close relative *U. urens* (annual, seed propagation) also translates into a higher root/shoot ratio in *U. dioica* compared to *U. urens.* The root fraction in *U. dioica* accounted for 20% of the total biomass in one-year-old plants compared to 5% in the annual *U. urens* [21]. Fertilization experiments with NO_3_ or NH_4_ alone indicated that nitrogen under the form of nitrate was preferred from ammonium for higher root growth. A low nitrogen supply favored the partitioning of biomass to the root system in greenhouse plants [22]. Different levels of NPK fertilization in a seasonal study showed that, with the exception of an early season, high nutrient levels led to a higher root biomass from August to December [20]. The number of new rhizomes was also affected by fertilization, with more and longer new rhizomes under higher fertilization.

Flooding is an abiotic stress that seriously affects nettle growth, and seedlings are especially sensitive to flooding in spring and summer. A container experiment with different water table depth (10–60 cm) showed that biomass, plant height, branching of stems and rhizomes, and rhizome length decreased in containers with a shallower water table depth [23]. The same study also showed that the number of rhizomes was correlated to the total above-ground biomass but not to plant height, whereas rhizome biomass was positively correlated to plant height and increased with water table depth.

## 3. Root Phytochemicals

With a few exceptions (e.g., [24,25]), available analyses were made from the whole root system, with unsorted roots and rhizomes. Although roots and rhizomes have different roles and their chemical composition may differ, as far we know, no data are available about the composition of roots versus rhizomes in nettle. As seen in Figure 1, the differentiation between roots and rhizomes might be difficult to establish.

The chemical composition of wild nettle roots collected in July before blooming was 13.66% crude protein, 1.65% crude fat, 31.65% crude fibers, 18.41% crude ash, 34.63% non-N compounds, and 2.17 mg/100 g DW carotenoids [11]. Roots collected earlier in the growing season had a protein content ranging from 3.31 to 10.89%, with a mean value of 6.74% DW [26].

**Figure 2:**
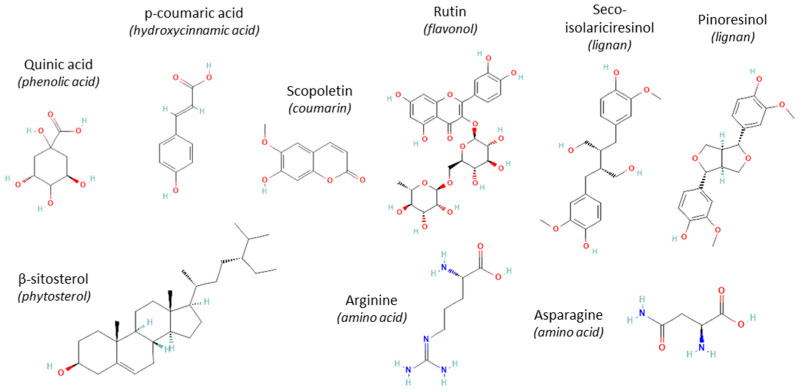
Representation of the most abundant soluble phytochemicals in nettle roots based on the literature. (Formula uploaded from PubChem https://pubchem.ncbi.nlm.nih.gov/ (accessed 9 January 2025).

Phytochemicals in nettle above- and underground organs were previously reported (e.g., [10]) but here, we focus exclusively on roots and add information regarding the material used, and extraction and detection methods. The stability of phytochemicals during past-harvest processing such as drying, different solubility in the extraction solvents, or detection methods are all factors affecting the final concentrations measured (e.g., [5,11,27,28]). The phytochemical composition of nettle roots is presented in Table 1 by compound type and publication in chronological order of the most important data. An analysis of commercial root extracts is not included. The most important phytochemicals are also shown in Figure 2. Only soluble phytochemicals are represented as they are the most easily extractable for possible applications. Lectins (UDA) are discussed in detail in the next section.

Roots are accumulating nitrogen reserves during summer and winter, mainly under the form of N-rich amino acids, asparagine and arginine [22]. Asparagine especially accumulates in young rhizomes and arginine in old rhizomes and roots [29]. Those stores are then sequentially mobilized and translocated in early spring to the upper plant parts for protein synthesis, enabling the shoot to undergo rapid growth prior to the development of their own capacity for nitrogen assimilation [25,29]. Arginine is known for its role as a nitrogen reserve in overwintering organs in some plants, mobilized at the onset of growth [29].

A similar change can be seen in the total phenolics. Changes in root total phenolics were measured using the Folin-Ciocalteu (FC) and ferric reducing ability of plasma (FRAP) analysis from water and ethanolic root extracts: a slight increase in values was measured at the end of the season in ethanolic extracts only, highlighting seasonal changes in root biochemical composition [5]. However, both tests report a similar type of antioxidant activity based on single electron transfer but measured at a different pH, and although commonly used as such, the FC is not specific to soluble phenolics [30]. However, a later study using HPLC showed higher concentrations of soluble phenolics in the spring compared to autumn samples (see Table 1, [27]).

**Table 1:** Phytochemicals in nettle roots. For each group of compounds, the data are listed in chronologic order of publication.

Plant Material	Methods	Results	Related Analysis, Comments	Reference
**Mineral**				
Dried roots (6 regions, Macedonia)	Atomic absorption spectrophotometry	Mg: 0.69–1.15% Ca: 0.61–0.92% Cu: 6.57–14.42 mg/kg Zn: 13.91–25.18 mg/kg Mn: 2.78–17.99 mg/kg Co: 0.08–0.16 mg/kg	Leaves and stems also analyzed.	[26]
**Phenolics**				
Dried roots	Supercritical extraction (SFE, ethanol in CO_2_), liquid–solid extraction with sonication (SAE, diethylether)	Scopoletin: 0.058 g/g DW (SFE) or 0.016 g/g DW (SAE)	Extraction yield increasing with increasing ethanol concentration in CO_2_. Yield decreasing with root storage time (matrix-binding)	[28]
Fresh frozen root (4 regions, Turkey)	80% methanol extraction, FC, DPPH antioxidant capacity, HPLC-DAD	FC: 20.12–1020.16 mg GAE/g DM DPPH: 30.60–370.27 mgGAE/g DW Quantification of flavonols (rutin as most abundant), hydroxycinnamic acids, ellagic acid, naringenin	Statistically significant difference between origins	[6]
Dried roots (3 locations, Serbia)	80% methanol extraction, HPLC-MS	Detected in all 3 sites (mg/g DW): Secoisolariciresinol (0–0.20), p-coumaric acid (0.12–0.23), quinic acid (0.1–0.36), (chlorogenic acid (0.025–0.056) and scopoletin (0.076–0.18) Others, minor: rutin, p-hydroxybenzoic, esculetin, caffeic acid, chrysoeriol, amentoflavone	Stems, leaves, and inflorescences also analyzed	[31]
Dried roots	80% methanol, LC-MS/MS	In decreasing abundance: quinic acid, p-coumaric acid, secoisolariciresinol (up to 0.04%), scopoletin	Aerial part analyzed as well, effect on inflammatory response	[32]
Freeze-dried roots (greenhouse plants, “clone 13”)	300 mM NaOH in 70% methanol, UPLC-Q-ToF	Lignans: pinoresinol most abundant (87.19 µg/g DW); pinoresinol diglucoside, lariciresinol and secoisolariciresinol (ca. 1 µg/g each)	Other tissues analyzed; expression of genes involved in biosynthesis	[33]
Dried root (Apr-Sep samples, Hungary)	Water (80 or 100 °C) or 96% ethanolic extracts (30 °C), FC, HPLC-DAD	Main phenolcarboxylic acids: vanillic, cinnamic, dihydroxy-benzoic, chlorogenic acids Main flavonoids: epicatechin, pyrocatechin, catechin, rutin, quercetin, pyrocatechin	Higher concentrations in spring. 5–10 times less polyphenolics than in leaves. Antimicrobial tests (bacteria, yeast)	[27]
**Terpenenoids**				
-	Methanol, ethyl acetate, GM-MS	2 major (0.85–1.1 mg/kg) + 1 minor monoterpene diols and their glucosides	Possibly products of enzymatic oxidation of α-pinene	[34]
**Phytosterols**				
Dried roots	Methanolic extract (5 day extraction), thin layer chromatograph, NMR.	β-sitosterol: 0.2–1% β-sitosterol-b-D-glucoside: 0.05–0.2% 2 sitosterol derivatives, 4 oxidation products: 2.0–5.6 mg/kg each	4 oxidation products (#5–8)	[35,36]
Dried roots	20% methanolic extract (ME20)	β-sitosterol: 0.49%	Antiproliferative activity on epithelial tumor cells of ME20 crude extract	[37]
Dried, flowering stage	Aqueous ethanolic extracts, IR	β-sitosterol: 0.03%		[38]
Dried roots	Supercritical extraction (SFE, ethanol in CO_2_), sonicated-assisted extraction (SAE, diethylether)	β-sitosterol: 0.63 mg/g DW (SFE) or 0.26 mg/g DW (SAE)	Yield independent of ethanol concentration in CO_2_	[28]
Freeze-dried root (October, 12 habitats, 3 regions, Croatia)	n-hexane extraction, GC-FID, GC-MS	Phytosterols: 0.80–0.86 g/kg DW (10 compounds identified, most abundant: β-sitosterol (74.65–81.68% of all sterols). Pentacyclic triterpenes: 0.0048–0.0070 g/kg DW (3 compounds detected, most abundant: β-amyrin acetate or β-amyrin)	No significant differences between regions	[39]
**Lipids and Fatty acids**				
Dried roots, (summer, Germany)	Methanol (8 weeks), TLC, GC-MS, IR, NMR	Two ceramide classes (1: 10 mg/kg, 2: 20 mg/kg) (E)-N-[2,3-dihydroxy-1- (hydroxymethyl)-7- heptadecenyl]] and its hydroxy-form (2-hydroxy fatty acid)	No aromatase inhibition activity	[40]
Freeze-dried roots (June, 5 locations, Spain)	Methanol, acetyl chloride, GC-FID (transesterification)	0.1 g/100 g DW Major compounds: C18:2 (34.3%), C16:0 (24%) and C18:1 (8.7%). (n − 3)/(n − 6) = 0.07		[41]
Wild, before blooming	n-hexane, GC-FID (NaOH saponification)	Saturated FA: 8.917% (most abundant: C18:0) Mono-unsaturated FA: 42.183% (most abundant: C18:1 (n − 9) cis+tr) Polyunsaturated: 48.90% (most abundant: C18:2 (n − 6), cis+tr)	Leaf FA profile also analyzed	[11]
**Lectins**				
Fresh frozen roots (winter, Belgium)	0.1 N HCl extract, Affinity chromatography on chitin	1 g/kg FW	Amino acid composition, Calculated MW 8.526 kDa Agglutination activity, induction of interferon in human lymphocytes	[42]
-		0.5–3.0 mg/g FW	Antifungal activities	[24]
Fresh and dried roots (winter, Germany)	0.1 N HCl extract, chitin affinity chromatography, ion exchange chromatography (IE), ELISA-HPLC	Two isolectins: 2.0% and 0.1% (DW)	UDA composition dependent on material and isolation strategy. Acidic polysaccharides, hemagglutination	[43]
Dried roots	20% methanolic extract (ME20)	0.03%	Antiproliferative activity on epithelial tumor cells of ME20 crude extract	[37]
**Amino acids**				
Dried roots, greenhouse plants at onset of flowering	N forms and free amino acid	Main free amino acids: Asn 16.5 µmol/g, GABA 12.5 µmol/g, Ala 6.5 µmol/g, Arg 1.7 µmol/g	Asparagine as main N storage form. *U. urens* also analyzed.	[21,29]
**Sugars**				
Dried roots, (winter, Germany)	Ethanol precipitate, GC, NMR	Acidic polysaccharides: 2 pectins (10 kda, 50 kDa), 2 rhamnogalacturanes (210 and 18 kDa), one type II arabinogalactan	UDA, hemagglutination	[43]
Dried roots	20% methanolic extract, GC-FID	Total: 10.23 mg/kg. Most abundant after hydrolysis were Glu (73.45%), followed by Galactose, Arabinose, Mannose, Rhamnose, Xylose	Proliferative activity of human prostatic epithelial (LNCaP)	[37]

## 4. UDA

Lectins are carbohydrate-binding proteins that bind glycans of glycoproteins, glycolipids, or polysaccharides with high affinity and act as agglutinins. Because of their binding specificity, they have the capability to serve as recognition molecules within a cell, between cells, or between organisms. They are found in different plant species and in many different organs and tissues where they play fundamental biological roles [44].

### 4.1. A Multigene Family/Protein Structure

**Figure 3:**
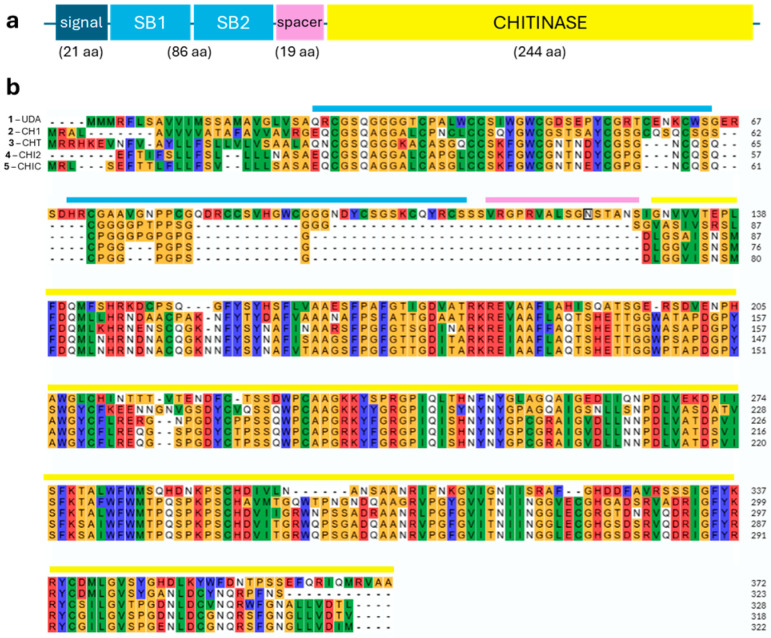
(**a**) UDA cDNA showing the structure of *uda1* sequence. Length of domains (sugar-binding domain, SB) are indicated as amino acid (aa) number [49]; (**b**) Nettle UDA1 (Uniprot entry P11218) aligned with 4 chitinase-containing sequences (CHI1—Q42993—*O. sativa*, CHIT—P05315—*S. tuberosum*, CHI3—P5240—*S. tuberosum*, CHIC—Q05538—*S. lycopersium*). The domains are indicated as in (**a**). The glycosylation site is boxed (N_123_). The alignment was made with Clustal Omega via Uniprot.

UDA is small (8.5 kDa), smaller than other plant lectins. It is stable in strong acidic conditions and can stand heat (10 min 85 °C) [42]. The first cDNA of UDA (*uda1*) was isolated by Lerner and Raihkhel in 1992 [45] using a synthetic probe. The cDNA encodes a protein of 21-amino acid putative signal sequence, 86 amino acids encoding the two chitin-binding domains characterized by eight cysteine residues, a 19-amino acid “spacer” domain, and a 244-amino acid carboxyl extension with partial similarity to a chitinase catalytic domain (Figure 3a) [45].

**Figure 4:**
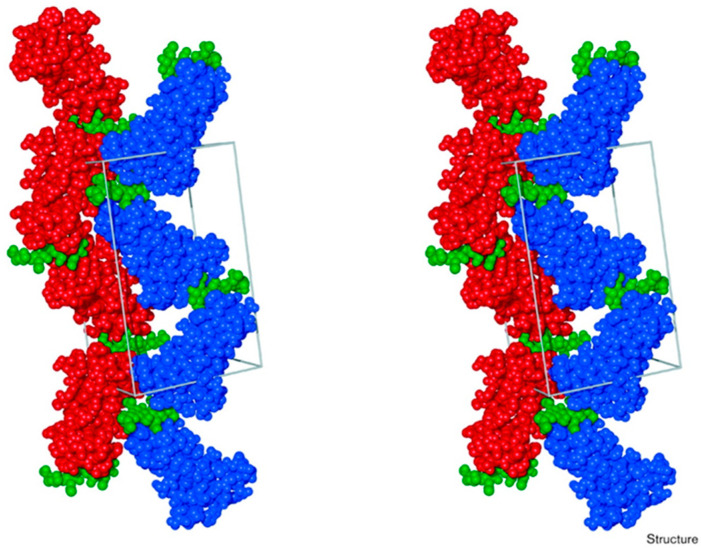
Crystal structure of UDA bound to (N, N′, N″, N‴ tetra-acetylchitotetraose) with gray lines delimiting the unit cell with UDA in red or blue and tetrasaccharide in green [51].

The protein belongs to the group of endochitinase I. In vivo, it is proteolytically processed to yield a small protein (8.5 kDa, 86 amino acids) containing only the two chitin-binding domains. The structure and sequence were later confirmed by the crystallization (Figure 4) of the UDA, showing two hevein-like motifs highly similar to wheat germ agglutinin [46]. Four disulfite bonds in each sugar-binding domain maintain the tertiary structure. The UDA is present as monomers or homodimers, with Zinc favoring dimerization [47]. Due to dimer formation, the sugar binding site of the C-terminal domain is blocked. The protein is, thus, likely active in the monomeric form but probably inactive in the dimeric form [47].

UDAs have similarities compared to other lectins, as identified on protein and genomic levels (Figure 3b) [42,48]. UDAs are part of a multigene family with at least 16 clones based on the Southern blotting and sequencing of cloned genes [45,49]. Alignment of the 16 available nettle isolectin sequences shows almost complete identity over the carbohydrate binding sites (Figure S2). With the recent publication of the nettle genome, we can see that all the UDA sequences from Does et al. 1999 [49] map into chromosome 11 of the assemblies of the two haplotypes [50]. UDA genomic localization can be seen in Supplementary Figure S3. Only three locations could be detected. Splicing variants or post-translational modifications may explain the 16 isoforms previously detected [49].

### 4.2. Spacio-Temporal Expression

Data on the expression of UDA genes are limited. At the plant level, van Damme and Peumans reported that the majority of the UDA protein localized in rhizomes, less in roots, and very little in stems [51]. Leaves did not contain any UDA. This was confirmed at the mRNA level by Lerner and Raikhel, who showed that the expression was limited to rhizomes and inflorescence buds (immature seeds) [45]. At the tissue level, UDA is present in the cortex of rhizomes and the outer exodermis cell layer of roots (unpublished data reported in [24]). The expression of the *uda1* gene in transgenic tobacco suggested a vacuolar localization of the wild type precursor protein [52]. The removal of the C-terminal 25 amino acids in a mutant protein led to the secretion of the protein into the intercellular space. This fragment, which does not align with other chitinase sequences, may contain a signal that retains UDA into the vacuole [52]. No data were found on the seasonal regulation of *uda* gene expression.

### 4.3. Extraction, Affinity of UDA, and Yield

Extraction of UDA from roots or rhizomes can be divided into three steps. Firstly, an acidic crude extract is prepared, containing a mixture of proteins from nettle roots. Secondly, the extract is cleaned by affinity chromatography with chitin or chitin-analogs to increase the UDA content. Thirdly, reverse phase chromatography is used to enrich and also separate the different iso-lectins [42,53]. The initial acidic extraction buffer preserves the activity of UDA, but steps 2 and 3 can be performed in more neutral buffers [42]. The affinity-based isolation provides good scalability, and the isolation does not require extensive amounts of harsh chemicals or solvents.

UDA affinity has been under study since the identification of the protein. Many of the isolation protocols leverage the innate affinity for chitin as a cheap and reliable method to isolate UDA from an acidic suspension [42,49]. Affinity for chitin supports the protective functionality of UDA for nettle. The sugar binding domains showed the highest affinities for the N,N’,N”-triacetylchitotriose unit [54]. More recently, affinities for other carbohydrate chains have been identified by using glycan microarrays [55,56]. The reports specifically focusing on UDA are compiled in Table 2. The studies showed that UDA has binding affinity to high-mannose-type N-glycan. It showed a relatively low, but significant affinity for mannose polymers, such as mannan derived from *Streptococcus aureus* and *Candida albicans*, which contrasts with other chitin-binding lectins [57]. More precisely, UDA recognizes Man3−Man9 and requires the chitobiose core. Chitin fragments (i.e., (−GlcNAcβ1,4GlcNAc−)n) and polymers of type 2 LacNAc (n ≥ 3 disaccharides) are also bound, but at a lower apparent affinity [58].

To have potential as a commercial product, UDA needs to be available in profitable amounts. The yield has not been well characterized in many of the assays or results are from only single isolations. The purity requirement for activity has not been addressed either. Yield of relatively pure UDA, purified with affinity chromatography, was between 0.3 and 6‰ (FW) from the starting material (Table 1). Optimized production could have higher yield if combined to induce the expression of UDA via chitin or insect exposure to the plant, but further optimization is required.

### 4.4. Chitinase Activity

Initial assumptions of the chitinase activity of UDA were based on the sequence similarity found by Lerner and Raikhel [45]. This activity was shown in vitro by the heterologous expression of uda1 (chi domain alone, full cDNA minus the putative signal sequence) and with a fusion protein expressing the chi domain [45,61]. High sequence similarity can be seen in Figure 3b with sequence comparison between UDA1 and 4 chitinase activity-containing proteins. The activity has also been shown with fusion proteins with other chitinase, which was shown to have affinity for protozoan *Phytomonas françai* [60]. In studies focusing on the native UDA, we found a different story. As native UDA was used as a control by R.A. Cole [62] to compare the activity of two brassica species lectins, UDA had similar activity compared to WGA and brassica lectins. But in the studies by Broekaert et al. (1989) [24], the extensively purified UDA from nettle roots shows no chitinase activity but keeps the agglutination and antifungal properties. Similar results have been found with bean lectin with the chitinase activity resulting from co-isolating proteins rather than from the lectin itself [24,63].

The chitinase story is not yet complete since the proteomic characterization of the final products and the visualization of the final isolated protein via gel electrophoresis could reveal the full characteristics of the isolate. It might be possible that the UDA still contains activity, but the protein requires processing or loses enzymatic activity during isolation, as suggested by Does et al. (1999) [52]. To fully understand the UDA family protein functionality, more research is required to accurately determine the functionality of different parts of the UDA amino acid sequence.

## 5. Pesticidal Activities

The functional activities of nettle root have been tested against different types of pests. Apart from UDA, data about the role of the individual phytochemicals in the activity are scarce. Crude extracts are usually tested, and their phytochemical composition is not always characterized. It is, therefore, challenging at that stage to associate activities with specific phytochemicals. The same lack of knowledge has been observed for health benefits (presented in the next section).

### 5.1. Antifungal and Antibacterial Activities

Nettle root extracts have been shown to have profound antifungal and antibacterial activity. Part of this activity comes from the chitin affinity of UDA, targeting the chitosan-containing glycans on the bacterial and fungal cell walls [24,52]. This was further confirmed by the lack of activity towards fungal and bacterial species without chitin in the cell wall [24]. The antifungal activity has been well characterized against *Botrytis cinerea* [24,52] where chitinase and UDA could act synergistically. UDA itself does not affect the spore germination but limits the hyphal growth by 85% [24]. The effect wastemporary, possibly due to an adaptation of the fungi cell wall [52]. Compared to *B. cinerea*, *T. viride* appeared to be more sensitive to UDA but had a later response time. The most important effects of UDA were observed for *C. lindemuthianum* [52]. It has been suggested that, because of its small size, UDA penetrates the cell wall where it affects cell wall synthesis [64].

Purified UDA and crude root extract also reduced hyphal growth of the mycorrhizal fungus *Glomus mosseae*, possibly explaining the inability of stinging nettle to form arbuscular mycorrhizal symbiosis with *G. mosseae* [65]. The occurrence of UDA at the periphery of the underground organs agrees with an activity against soil microbes [24].

Root water or ethanol extracts showed stronger antibacterial efficacy than leaves or stems extracts. Gram-negative bacteria (*P. vullgaris* and *K. pneumoniae*) were more susceptible than Gram-positive bacteria to the water extract of root in all the dilutions tested [66]. *Enterococcus faecalis* was considerably inhibited by aqueous root extracts, with spring or autumn root samples showing identical activity [27]. Root methanolic extract had antimicrobial activity against *Xanthomonas vesicatoria* (MIC = 1024 μg/mL) [67]. A comparison of the leaf, stem, and root extracts of *U. dioica* showed that non-polar extracts of the root were the most efficient against both Gram-positive (*Staphylococcus aureus*, *Pseudomonas aeruginosa*) and Gram-negative bacteria (*Bacillus subtilis*, *E. coli*) at the concentration used (6.25 mg/mL) [68].

### 5.2. Antiviral Activity

UDA was demonstrated to have antiviral activity in vitro towards a broad antiviral spectrum. Studies report activity against SARS- and SARS-CoV-2, influenza A and B viruses, Dengue virus serotype 2 (DENV-2) and HIV, herpes simplex virus type 1 (HSV-1) and HSV-2, varicella zoster virus (VZV), and parainfluenza virus [69,70,71,72]. However, it did not display any antiviral activity to non-enveloped viruses such as coxsackie virus and reovirus [69].

We found no publications focusing on the antiviral activity of nettle lectins on plant viruses. However, UDA1, even being from another lectin family, shares sequence similarity with both JAX1 and RTM1, which are shown to have protective activity against the mosaic virus infection [73]. The antiviral activity of UDA could be a result of the binding affinity and agglutination binding to the surface proteins on the viral capsid, as shown for the viral spike protein of SARS-CoV-2 [72].

### 5.3. Insecticidal Activity

Although the raw material and UDA extraction method were not described in their report, Cole and coworkers showed a significant reduction in cabbage aphid *Brevicoryne brassicae* survival when fed lectin-supplemented diets [62]. Insecticidal effects for concentrations higher than 25 µg/mL UDA were observed.

## 6. Health Benefits

The health benefits of the aerial part but also the roots of nettle have been recently reviewed extensively [15,16,74,75,76,77] and thus, the medicinal applications of root extracts will only be briefly listed here. The readers are invited to consult the above-mentioned reviews or cited studies for further details.

The best-known and most described application of nettle root is the treatment of lower urinary tract symptoms (LUTSs) due to prostate benign hyperplasia (BHP) [74]. The effects have been demonstrated in many in vitro but also clinical assays [78,79,80]. Stinging nettle, recommended for LUTS management, is in the category of traditional use, which means that it does not fulfill the requirements for marketing authorization, but there are sufficient safety data and plausible efficacy based on longstanding use and experience. Phytosterols (β-sitosterol), lignans, polysaccharides, and the lectin UDA are considered to be among the active principles, but the active role of each compound is not fully demonstrated [16,74,76,77].

Nettle root extracts have shown antiproliferative effects against benign but also malicious tumorous cells [36,77]. UDA has cytotoxic and apoptotic effects on acute myeloid leukemia human cells, where the inhibition of cell proliferation and the induction of apoptosis was dose-dependent, and the expression of the caspase-9 dependent pathway was increased [81]. Hydroalcoholic root extracts inhibited the proliferation of human gastric and colorectal cancer cells, with effects comparable to oxaliplatin, a standard chemotherapy drug [82].

Although nettle root extracts have low cytotoxicity, some side effects have been discussed. In rats, spermatogenesis and spermiogenesis following the consumption of *U. dioica* root extract was reduced [83].

Several studies report the activity of nettle root extracts on the immune system. T lymphocytes are activated in a dose-dependent manner by purified UDA [43] and UDA was referred to as a superantigen for T cell activation [84]. A database of 277 nettle phytochemicals was screened for their potential efficiency in treating allergic rhinitis by in silico evaluation [85]. Interestingly, several root-specific compounds such as sitosterol and its derivatives showed high affinity against known therapeutic targets of allergic rhinitis. This result would confirm the earlier clinical trial results about root extract efficiency in treating rhinitis [86].

Among the other beneficial health effects of root extracts, one can mention metabolic control [87,88] and cardiovascular effects [89].

## 7. Conclusions and Future Directions

In general, nettle roots represent a valuable raw material and their market is expected to increase rapidly in relation to the high consumer demand for herbal and natural supplements. Although capsules of dried ground nettle roots are the most common form found in the market, purified compounds, especially UDA, have a bright future due to their chemical and functional properties (health benefits, plant protection applications).

However, several gaps in knowledge can be identified on the function, composition, and phytochemical functional activities of nettle roots. Here, we propose some research needs, with their potential benefits in term of applications in different industrial sectors, summarized in Table 3.

In addition to the major and best characterized phytochemicals (sitosterol and derivatives, scopoletin, UDA, lignans, polysaccharides), a lot of variation in the composition of minor compounds is observed (Table 1). The origin of the raw material (genetic background, growing conditions, sampling time), but also the extraction and analyses methods, are all factors that can explain this variability. In a recent review by Harrison et al. about the antibacterial activity of stinging nettle extract [90], a literature screening excluded 298 of 313 publications from the initial dataset with missing information, duplicates, or a lack of replicate results. This shows the limitations in the experiments performed with nettle extracts and demonstrates the need for a standardized approach to reveal the active components for antifungal and antibacterial applications.

Results diverge when considering the effect of the sampling site, with significant [6] or non-significant [39] effects. High variability was measured in the mineral or phenolic content of roots [26,31]. Strong inter-individual variation was also reported for lectin composition [91]. As a comparison, rather similar phenolic profiles were observed in leaves from different origins [17,92].

Generally, data about the seasonal variation of root phytochemical composition are scarce. As for any other plant material, seasonality and environmental conditions should be taken into consideration for the valorization of the raw material [5,28,39].

UDAs are particularly interesting for many applications. Although UDA purification can be rather cost-efficient, the supply of nettle likely still needs optimization (see below). As far we know, the question about the chitinase activity of UDA remains open. Surprisingly, no information has been published about UDA expression control recently. The functional activity of UDA, in combination with its heat stability, makes it an interesting raw material for the production of phytosanitary products.

Globally, there is a large gap in knowledge on the regulation of phytochemical synthesis in nettle roots. Different levels can be considered.

The genetic background of the raw material, as discussed above, is the first level. Easier access to DNA-based identification methods and the recent sequencing of the nettle genome [50] should facilitate a more precise identification of the raw material.

The regulation of the biosynthetic gene expression is the second level. The expression of the lignan biosynthetic genes has been studied in roots [33]. On the other hand, UDAs are encoded by a multigene family [49], but as far as is known, no information is available about the role and regulation of the different *uda* genes. Control by internal (hormones, tissue, age) but also external factors (growing conditions, fertilization, abiotic or biotic stress) in root tissues requires further research. To our knowledge, no detailed data are available about the seasonal expression of the UDA multigene family, nor the possible response to biotic stress if UDA is considered as a pathogenesis-related protein (putative chitinase activity). In nettle leaves, drought was tested to increase the vitamin C content, phenolics, and pigments [93].

The third level targets the production of roots as raw material. Harvesting field-grown nettle roots is laborious but also deleterious to future harvest. Many factors can affect the concentration of targeted phytochemicals during harvesting, washing, and sorting. Controlled-environment agriculture techniques would counter many of these issues by producing standardized quality raw material. Hydroponic or aeroponic cultivation methods are appealing approaches for the year-round biomass production of standardized raw material. It was used for the production of high-value medicinal plants [94] and the controlled growing conditions allows for increasing the concentrations of bioactive molecules [95,96]. Hydroponic or aeroponic cultivation technics are well adapted to nettle [97,98,99]. In addition to the root, the young green shoots produced are premium raw material and could readily be used in food or medicinal applications [15,98]. Nettle is rapidly accumulating N under the form of nitrate, primarily in stems and petiole [21]. The NO_3_ content in young leaves from aeroponically cultivated nettle was closely correlated with the nutrient solution composition and was rapidly exceeding the accepted limit for food applications (Martz, unpublished, in collaboration with Lapland University of Applied Science), so optimized growing conditions are needed. The expected root biomass, frequency of pruning, root regrowth dynamic, and effect on the aerial part after pruning, are all areas that need to be addressed to evaluate the potential of the hydroponic or aeroponic production of nettle roots. Finally, cell culture must be mentioned as well as another promising approach to produce plant-based high value phytochemicals.

Despite several gaps in knowledge, we hope the readers will be convinced of the potential of stinging nettle roots as a source of active biomolecules in the pharmaceutical and agricultural industries.

## Figures and Tables

**Table 2:** UDA affinity based on lectin or glycan microarray analysis. (M: mannose).

Reference	Method	Main Results, Comment
[59]	Lectin microarray. Fluorescent detection with tetramethylrhodamine-labeled oligosaccharides	UDA is considered to recognize multiple sites of the core mannosyl structure (Man3-GlcNAc2) so that a simple monosaccharide, mannose, cannot exert sufficient power of inhibition at the given concentration (10 mM). This was sufficient to compete with another mannose-specific lectin (Concanavalin-A) bound tetramethylrhodamine -M6 showing UDA’s higher affinity towards the TMR-Man6 structure.
[60]	Glycan array combined with computational modeling.	UDA binding was characterized via minimal binding determinant datasets and computational modeling. UDA: Asp26, Ser27, Arg16, Tyr30 bind two different oligosaccharides.
[57]	Frontal affinity chromatography (FAC) and glycan array.	Isolectin UDA6 binding via tandem-repeat type of sequence. Molecular kinetics and dissociation constants measured against specific oligosaccharides. The use of extensive glycan panel (124 glycans) allowed for assessing the affinity of lectins. UDA had a higher affinity towards high-mannose-type N-glycan polymers (affinity increased with the number of mannose residues).
[58]	Glycan array screening.	Machine learning and utilization of available open dataset of Consortium for Functional Glycomics glycan microarray (CFGv5). UDAs bind to mannose-containing chitobiose chains, N-acetyllactosamine, and chitin fragments.

**Table 3:** Proposed needs in nettle root research and their potential benefits in terms of applications in different industrial sectors.

Topic	Research Needs	Benefits/Applications (*Industry Sector*)
Phytochemical abundance	Genetic background. Control of biosynthesis pathways by, for example, nutrients, environmental conditions (temperature, edaphic factors), abiotic and biotic stress, seasonality.	Selection of best nettle accession and optimization of harvesting time; possibility to stimulate the biosynthesis of targeted phytochemicals (*all: pharmaceuticals, agriculture*).
Activity of phytochemicals/crude extracts	Chemical characterization of crude extracts; role of individual phytochemicals in activity.	Optimization of the extraction conditions and production of concentrated extracts; e.g., BPH treatment, anticancer (*pharmaceuticals*).
UDA	Protein maturation: fate of the chitinase sequence and its activity.	Antifungal, antibacterial, antiviral active biomolecules (*agriculture, pharmaceuticals*).
Controlled environment agriculture (hydroponic, aeroponic, cell culture)	Optimization of cultivation conditions (e.g., nutrient supply (N), light) and phytochemical composition.	Standardized and clean root raw material supply (*pharmaceutics, agriculture*), possibly combined with premium leaf biomass production (*food, nutraceuticals, pharmaceuticals*).

## Supplementary Materials

The following supporting information can be downloaded at: https://www.mdpi.com/article/10.3390/plants14020279/s1, Figure S1: Number of articles related to nettle roots, organized by publication year (a) and countries/year (b); Figure S2: Alignment of nucleotide sequences of UDA-isoforms from Does et al. (1999) [49]; Figure S3: Location of UDA-sequences from Does et al 1999 (N-ambiguities removed) in the new nettle genome assembly [50].
