# Supplementary material for: Stinging Nettle (Urtica dioica) Roots: The Power Underground—A Review
Source: Plants (Basel). 2025 Jan 19;14(2):279. doi: 10.3390/plants14020279 (PMC11768490; doi:10.3390/plants14020279)
Supplement: Supplementary file 1 [file plants-14-00279-s001.zip › plants-3411955-supplementary.pdf]

Martz and Kankaanpää: Stinging nettle (*Urtica dioica*) roots: the power underground - a review

Supplementary figures

Contents

Figure S1 Number of articles related to nettle roots, organized by publication year (a) and countries/year (b). 1

Figure S2 Alignment of nucleotide sequences of UDA-isoforms from Does et al. (1999) [49]. .2

Figure S3 Location of UDA-sequences from Does et al 1999 (N-ambiguities removed) in the new nettle genome assembly [50]. 8

Figure S1 Number of articles related to nettle roots, organized by publication year (**a**) and countries/year (**b**).
N=82 (Jan 2025), general reviews or articles about nettle other than roots were excluded. In b, one dot can represent more than one article.

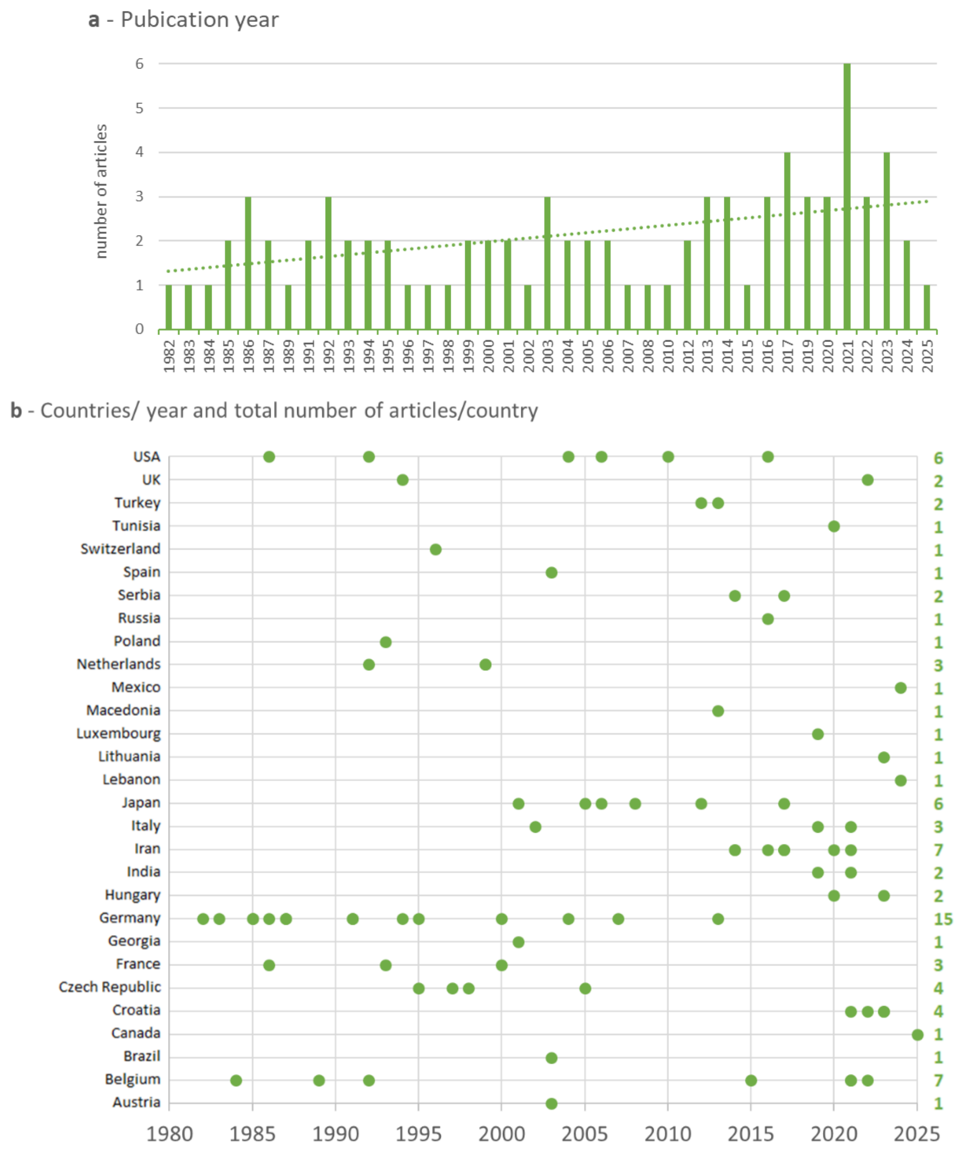

Figure S2 Alignment of nucleotide sequences of UDA-isoforms from Does et al. (1999) [49]. Does et al. 1999: https://link.springer.com/article/10.1023/A:1006134932290. *The initiation codon ATG is highlighted in yellow. N: base not characterized.*

```
Consensus                 AATCATAGTAAGAAAGAAAAGATGATGATGAGGTTTTTAGCTGCCGTAGTGATCATGTCC   60
ENA|AF059535|AF059535.1 AATCATAGTAAGAAAGAAAAGATGATGATGAGGTTTTTAGCTGCCGTAGTGATCATGTCC   60
AH007417.2                ---------------------ATGATGATGAGGTTTTTAGCTGCCGTAGTGATCATGTCC   39
AH007416.2                ---------------------ATGATGATGAGGTTTTTAGCTGCCGTAGTGATCATGTCC   39
AH007415.2                ---------------------ATGATGATGAGGTTTTTAGCTGCCGTAGTGATCATGTCC   39
AH007265.2                ---------------------ATGATGATGAGGTTTTTAGCTGCCGTAGTGATCATGTCC   39
AH007264.2                ---------------------ATGATGATGAGGTTTTTATCTGCCGTAGTGATCATGTCC   39
AH007263.2                ---------------------ATGATGATGAGGTTTTTATCTGCCGTAGTGATCATGTCC   39
AH007228.2                ---------------------ATGATGATGAGGTTTTTAGCTGCCGTAGTGATCATGTCC   39
AH007227.2                ---------------------ATGATGATGAGGTTTTTAGCTGCCGTAGTGATCATGTCC   39
AH007223.2                ---------------------ATGATGATGAGGTTTTTAGCTGCCGTAGTGATCATGTCC   39
AH007222.2                ---------------------ATGATGATGAGGTTTTTAGCTGCCGTAGTGATCATGTCC   39
AH007221.2                ---------------------ATGATGATGAGGTTTTTAGCTGCCGTAGTGATCATGTCC   39
AH007220.2                ---------------------ATGATGATGAGGTTTTTAGCTGCCGTAGTGATCATGTCC   39
AH007219.2                ---------------------ATGATGATGAGGTTTTTATCTGCCGTAGTGATCATGTCC   39
AH007218.2                ---------------------ATGATGATGAGGTTTTTATCTGCCGTAGTGATCATGTCC   39
AH007217.2                ---------------------ATGATGATGAGGTTTTTAGCTGCCGTAGTGATCATGTCC   39

Consensus                 TCCGCTATGGCGGTGGGTCTAGTGTCGGCA---CAGAGGTGCGGAAGCCAAGGCGGCGGG   117
ENA|AF059535|AF059535.1 TCCGCTATGGCGGTGGGTCTAGTGTCGGCA---CAGAGGTGCGGAAGCCAAGGCGGCGGG   117
AH007417.2                TCCGCTATGGCGGTGGGTCTAGTGTCGGCA---CAGAGGTGCGGAAGCCAAGGCGGCGGG   96
AH007416.2                TCCGCTATGGCGGTGGGTCTAGTGTCGGCA---CAGAGGTGCGGAAGCCAAGGCGGCGGG   96
AH007415.2                TCCGCTATGGCGGTGGGTCTAGTGTCGGCA---CAGAGGTGCGGAAGCCAAGGCGGCGGG   96
AH007265.2                TCCGCTATGGCGGTGGGTCTAGTGTCGGCA---CAGAGGTGCGGAAGCCAAGGCGGCGGG   96
AH007264.2                TCCGCTATGGCGGTGGGTCTAGTGTCGGCA---CAGAGGTGCGGAAGCCAAGGCGGCGGG   96
AH007263.2                TCCGCTATGGCGGTGGGTCTAGTGTCGGCA---CAGAGGTGCGGAAGCCAAGGCGGCGGG   96
AH007228.2                TCCGCTATGGCGGTGGGTCTAGTGTCGGCA---CAGAGGTGCGGAAGCCAAGGCGGCGGG   96
AH007227.2                TCCGCTATGGCGGTGGGTCTAGTGTCGGCA---CAGAGGTGCGGAAGCCAAGGCGGCGGG   96
AH007223.2                TCCGCTATGGCGGTGGGTCTAGTGTCGGCA---CAGAGGTGCGGAAGCCTAGGCGGCGGG   96
AH007222.2                TCCGCTATGGCGGTGGGTCTAGTGTCGGCA---CAGAGGTGCGGAAGCCTAGGCGGCGGG   96
AH007221.2                TCCGCTATGGCGGTGGGTCTAGTGTCGGCACAGCAGAGGTGCGGAAGCCAAGGCGGCGGG   99
AH007220.2                TCCGCTATGGCGGTGGGTCTAGTGTCGGCACAGCAGAGGTGCGGAAGCCAAGGCGGCGGG   99
AH007219.2                TCCGCTATGGCGGTGGGTCTAGTGTCGGCA---CAGAGGTGCGGAAGCCAAGGCGGCGGG   96
AH007218.2                TCCGCTATGGCGGTGGGTCTAGTGTCGGCA---CAGAGGTGCGGAAGCCAAGGCGGCGGG   96
AH007217.2                TCCGCTATGGCGGTGGGTCTAGTGTCGGCA---CAGAGGTGCGGAAGCCAAGGCGGCGGG   96

Consensus                 GGTACGTGTCCCGSCTTGYGGTGCTGCAGCATCTGGGGCTGGTGCGGCGACTCGGAGCCC   177
ENA|AF059535|AF059535.1 GGTACGTGTCCCGCCTTGTGGTGCTGCAGCATCTGGGGCTGGTGCGGCGACTCGGAGCCC   177
AH007417.2                GGTACGTGTCCCGGCTTGCGGTGCTGCAGCATCTGGGGCTGGTGCGGCGACTCGGAGCCC   156
AH007416.2                GGTACGTGTCCCGGCTTGCGGTGCTGCAGCATCTGGGGCTGGTGCGGCGACTCGGAGCCC   156
AH007415.2                GGTACGTGTCCCGGCTTGCGGTGCTGCAGCATCTGGGGCTGGTGCGGCGACTCGGAGCCC   156
AH007265.2                AGTACGTGTCCCGGCTTGCGGTGCTGCAGCATCTGGGGCTGGTGCGGCGACTCGGAGCCC   156
AH007264.2                GGTACGTGTCCCGGCTTGCGGTGCTGCAGCATCTGGGGCTGGTGCGGCGACTCGGAGCCC   156
AH007263.2                GGTACGTGTCCCGGCTTGCGGTGCTGCAGCATCTGGGGCTGGTGCGGCGACTCGGAGCCC   156
AH007228.2                GGTACGTGTCCTGCCTTGTGGTGCTGCAGCATCTGGGGCTGGTGCGGCGACTCGGAGCCC   156
AH007227.2                GGTACGTGTCCCGCCTTGTGGTGCTGCAGCATCTGGGGCTGGTGCGGCGACTCGGAGCCC   156
AH007223.2                GGTACGTGTCCCGGCTTGCGGTGCTGCAGCATCTGGGGCTGGTGCGGCGACTCGGAGCCC   156
AH007222.2                GGTACGTGTCCCGGCTTGCGGTGCTGCAGCATCTGGGGCTGGTGCGGCGACTCGGAGCCC   156
AH007221.2                GGTACGTGTCCCGCCTTGTGGTGCTGCAGCATCTGGGGCTGGTGCGGCGACTCGGAGCCC   159
AH007220.2                GGTACGTGTCCCGCCTTGTGGTGCTGCAGCATCTGGGGCTGGTGCGGCGACTCGGAGCCC   159
AH007219.2                GGTACGTGTCCTGCCTTGTGGTGCTGCAGCATCTGGGGCTGGTGCGGCGACTCGGAGCCC   156
AH007218.2                GGTACGTGTCCTGCCTTGTGGTGCTGCAGCATCTGGGGCTGGTGCGGCGACTCGGAGCCC   156
AH007217.2                GGTACGTGTCCTGCCTTGTGGTGCTGCAGCATCTGGGGCTGGTGCGGCGACTCGGAGCCC   156

Consensus                 TACTGCGGCCGCACCTGCGAGAACAAGTGCTGGAGCGGCGAGCGGTCGGACCACCGCTGC   237
ENA|AF059535|AF059535.1 TACTGCGGCCGCACCTGCGAGAACAAGTGCTGGAGCGGCGAGCGGTCGGACCACCGCTGC   237
AH007417.2                TACTGCGGCCGCACCTGCGAGAACAAGTGCTGGAGCGGCGAGAGGTCGGACCACCGCTGC   216
AH007416.2                TACTGCGGCCGCACCTGCGAGAACAAGTGCTGGAGCGGCGAGAGGTCGGACCACCGCTGC   216
AH007415.2                TACTGCGGCCGCACCTGCGAGAACAAGTGCTGGAGCGGCGAGAGGTCGGACCACCGCTGC   216
AH007265.2                TACTGCGGCCGCACCTGCGAGAACAAGTGCTGGAGCGGCGAGAGGTCGGACCACCGCTGC   216
AH007264.2                TACTGCGGCCGCACCTGCGAGAACAAGTGCTGGAGCGGCGAGAGGTCGGACCACCGCTGC   216
AH007263.2                TACTGCGGCCGCACCTGCGAGAACAAGTGCTGGAGCGGCGAGAGGTCGGACCACCGCTGC   216
AH007228.2                TACTGCGGCCGCACCTGCGAGAACAAGTGCTGGAGCGGCGAGCGGTCGGACCACCGCTGC   216
AH007227.2                TACTGCGGCCGCACCTGCGAGAACAAGTGCTGGAGCGGCGAGCGGTCGGACCACCGCTGC   216
AH007223.2                TACTGCGGCCCCAGCTGCGAGACCAACTGCTGGGACGACGAGCGGTCCGACCACCGCTGC   216
AH007222.2                TACTGCGGCCCCAGCTGCGAGACCAACTGCTGGGACGACGAGCGGTCCGACCACCGCTGC   216
AH007221.2                TACTGCGGCCGCACCTGCGAGAACAAGTGCTGGAGCGGCGAGCGGTCGGACCACCGCTGC   219
AH007220.2                TACTGCGGCCGCACCTGCGAGAACAAGTGCTGGAGCGGCGAGCGGTCGGACCACCGCTGC   219
AH007219.2                TACTGCGGCCGCACCTGCGAGAACAAGTGCTGGAGCGGCGAGCGGTCGGACCACCGCTGC   216
AH007218.2                TACTGCGGCCGCACCTGCGAGAACAAGTGCTGGAGCGGCGAGCGGTCGGACCACCGCTGC   216
AH007217.2                TACTGCGGCCGCACCTGCGAGAACAAGTGCTGGAGCGGCGAGCGGTCGGACCACCGCTGC   216

Consensus                 GGCGCCGCTGTMGGAAACCCTCCGTGCGGCCAGGACCGGTGCTGCAGCGTCCACGGGTGG   297
ENA|AF059535|AF059535.1 GGCGCCGCTGTAGGAAACCCTCCGTGCGGCCAGGACCGGTGCTGCAGCGTCCACGGGTGG   297
```

```
AH007417.2              GGCGCCGCTGTCGGAAACCCTCCGTGCGGCAAGGACCGGTGCTGCAGCGTCCACGGGTGG   276
AH007416.2              GGCGCCGCTGTCGGAAACCCTCCGTGCGGCAAGGACCGGTGCTGCAGCGTCCACGGGTGG   276
AH007415.2              GGCGCCGCTGTCGGAAACCCTCCGTGCGGCAAGGACCGGTGCTGCAGCGTCCACGGGTGG   276
AH007265.2              GGCGCCGCTGTCGGAAACCCTCCGTGCGGCCAGGACCGGTGCTGCAGCGTCCACGGGTGG   276
AH007264.2              GGCGCCGCTGTCGGAAACCCTCCGTGCGGCCAGGACCGGTGCTGCAGCGTCCACGGGTGG   276
AH007263.2              GGCGCCGCTGTCGGAAACCCTCCGTGCGGCCAGGACCGGTGCTGCAGCGTCCACGGGTGG   276
AH007228.2              GGCGCCGCTGTAGGAAACCCTCCGTGCGGCCAGGACCGGTGCTGCAGCGTCCACGGGTGG   276
AH007227.2              GGCGCCGCTGTAGGAAACCCTCCGTGCGGCCAGGACCGGTGCTGCAGCGTCCACGGGTGG   276
AH007223.2              GGCGCCGCTGTCGGAAACCCTCCGTGCGGCCAGGACCGGTGCTGCAGCGTCCACGGGTGG   276
AH007222.2              GGCGCCGCTGTCGGAAACCCTCCGTGCGGCCAGGACCGGTGCTGCAGCGTCCACGGGTGG   276
AH007221.2              GGCGCCGCTGTAGGAAACCCTCCGTGCGGCCAGGACCGGTGCTGCAGCGTCCACGGGTGG   279
AH007220.2              GGCGCCGCTGTAGGAAACCCTCCGTGCGGCCAGGACCGGTGCTGCAGCGTCCACGGGTGG   279
AH007219.2              GGCGCCGCTGTAGGAAACCCTCCGTGCGGCCAGGACCGGTGCTGCAGCGTCCACGGGTGG   276
AH007218.2              GGCGCCGCTGTAGGAAACCCTCCGTGCGGCCAGGACCGGTGCTGCAGCGTCCACGGGTGG   276
AH007217.2              GGCGCCGCTGTAGGAAACCCTCCGTGCGGCCAGGACCGGTGCTGCAGCGTCCACGGGTGG   276

Consensus               TGCGGTGGCGGCAACGACTACTGCTCCGGGAGCAAATGCCAGTACCGCTGCTCCTCCTCC   357
ENA|AF059535|AF059535.1 TGCGGTGGCGGCAACGACTACTGCTCCGGGAGCAAATGCCAGTACCGCTGCTCCTCCTCC   357
AH007417.2              TGCGGTGGCGGCAACGACTACTGCTCCGGGGGAAAATGCCAGTACCGCTGCTCCTCCTCC   336
AH007416.2              TGCGGTGGCGGCAACGACTACTGCTCCGGGGGAAAATGCCAGTACCGCTGCTCCTCCTCC   336
AH007415.2              TGCGGTGGCGGCAACGACTACTGCTCCGGGGGAAAATGCCAGTACCGCTGCTCCTCCTCC   336
AH007265.2              TGCGGTGGCGGCAACGACTACTGCTCCGGGGGCAACTGCCAGTACCGCTGCTCCTCCTCC   336
AH007264.2              TGCGGTGGCGGCAACGACTACTGCTCCGGGGGCAACTGCCAGTACCGCTGCTCCTCCTCC   336
AH007263.2              TGCGGTGGCGGCAACGACTACTGCTCCGGGGGCAACTGCCAGTACCGCTGCTCCTCCTCC   336
AH007228.2              TGCGGTGGCGGCAACGACTACTGCTCCGGGAGCAAATGCCAGTACCGCTGCTCCTCCTCC   336
AH007227.2              TGCGGTGGCGGCAACGACTACTGCTCCGGGAGCAAATGCCAGTACCGCTGCTCCTCCTCC   336
AH007223.2              TGCGGTGGCGGCTGGGACTACTGCTTCGGGGAAAACTGCCAGTACCGCTGCTCCTCCTCC   336
AH007222.2              TGCGGTGGCGGCAACGACTACTGCTC------------------------CTCCTCCNNN   312
AH007221.2              TGCGGTGGCGGCAACGACTACTGCTCCGGGAGCAAATGCCAGTACCGCTGCTCCTCCTCC   339
AH007220.2              TGCGGTGGCGGCAACGACTACTGCTCCGGGAGCAAATGCCAGTACCGCTGCTCCTCCTCC   339
AH007219.2              TGCGGTGGCGGCAACGACTACTGCTCCGGGAGCAAATGCCAGTACCGCTGCTCCTCCTCC   336
AH007218.2              TGCGGTGGCGGCAACGACTACTGCTCCGGGAGCAAATGCCAGTACCGCTGCTCCTCCTCC   336
AH007217.2              TGCGGTGGCGGCAACGACTACTGCTCCGGGAGCAAATGCCAGTACCGCTGCTCCTCCTCC   336

Consensus               GTCCGTG-----------------------------------------------------   364
ENA|AF059535|AF059535.1 GTCCGTGGACCCCGCGTCGCTCTCAGCGGCAATTCCACCGCCAACTCCATCGGCAACGTC   417
AH007417.2              NNNNNNN-----------------------------------------------------   343
AH007416.2              NNNNNNN-----------------------------------------------------   343
AH007415.2              NNNNNNN-----------------------------------------------------   343
AH007265.2              NNNNNNN-----------------------------------------------------   343
AH007264.2              NNNNNNN-----------------------------------------------------   343
AH007263.2              NNNNNNN-----------------------------------------------------   343
AH007228.2              NNNNNNN-----------------------------------------------------   343
AH007227.2              NNNNNNN-----------------------------------------------------   343
AH007223.2              NNNNNNN-----------------------------------------------------   343
AH007222.2              NNNNNNN-----------------------------------------------------   319
AH007221.2              NNNNNNN-----------------------------------------------------   346
AH007220.2              NNNNNNN-----------------------------------------------------   346
AH007219.2              NNNNNNN-----------------------------------------------------   343
AH007218.2              NNNNNNN-----------------------------------------------------   343
AH007217.2              NNNNNNN-----------------------------------------------------   343

Consensus               ------------------------------------------------------------   364
ENA|AF059535|AF059535.1 GTCGTCACCGAGCCGCTGTTCGACCAGATGTTCTCCCACCGCAAGGACTGTCCGAGCCAG   477
AH007417.2              ------------------------------------------------------------   343
AH007416.2              ------------------------------------------------------------   343
AH007415.2              ------------------------------------------------------------   343
AH007265.2              ------------------------------------------------------------   343
AH007264.2              ------------------------------------------------------------   343
AH007263.2              ------------------------------------------------------------   343
AH007228.2              ------------------------------------------------------------   343
AH007227.2              ------------------------------------------------------------   343
AH007223.2              ------------------------------------------------------------   343
AH007222.2              ------------------------------------------------------------   319
AH007221.2              ------------------------------------------------------------   346
AH007220.2              ------------------------------------------------------------   346
AH007219.2              ------------------------------------------------------------   343
AH007218.2              ------------------------------------------------------------   343
AH007217.2              ------------------------------------------------------------   343

Consensus               ------------------------------------------------------------   364
ENA|AF059535|AF059535.1 GGCTTCTACAGCTACCACTCCTTCCTCGTAGCCGCCGAGTCCTTCCCAGCTTTCGGGACC   537
AH007417.2              ------------------------------------------------------------   343
AH007416.2              ------------------------------------------------------------   343
AH007415.2              ------------------------------------------------------------   343
AH007265.2              ------------------------------------------------------------   343
AH007264.2              ------------------------------------------------------------   343
AH007263.2              ------------------------------------------------------------   343
AH007228.2              ------------------------------------------------------------   343
AH007227.2              ------------------------------------------------------------   343
AH007223.2              ------------------------------------------------------------   343
```

```
AH007222.2                 ------------------------------------------------------------   319
AH007221.2                 ------------------------------------------------------------   346
AH007220.2                 ------------------------------------------------------------   346
AH007219.2                 ------------------------------------------------------------   343
AH007218.2                 ------------------------------------------------------------   343
AH007217.2                 ------------------------------------------------------------   343

Consensus                  ------------------------------------------------------------   364
ENA|AF059535|AF059535.1 ATCGGAGATGTTGCGACACGCAAGAGAGAGGTCGCAGCGTTCCTCGCCCATATCTCCCAA   597
AH007417.2                 ------------------------------------------------------------   343
AH007416.2                 ------------------------------------------------------------   343
AH007415.2                 ------------------------------------------------------------   343
AH007265.2                 ------------------------------------------------------------   343
AH007264.2                 ------------------------------------------------------------   343
AH007263.2                 ------------------------------------------------------------   343
AH007228.2                 ------------------------------------------------------------   343
AH007227.2                 ------------------------------------------------------------   343
AH007223.2                 ------------------------------------------------------------   343
AH007222.2                 ------------------------------------------------------------   319
AH007221.2                 ------------------------------------------------------------   346
AH007220.2                 ------------------------------------------------------------   346
AH007219.2                 ------------------------------------------------------------   343
AH007218.2                 ------------------------------------------------------------   343
AH007217.2                 ------------------------------------------------------------   343

Consensus                  ------------------------------------------------------------   364
ENA|AF059535|AF059535.1 GCAACATCAGGTAGTATACTATCCCTTCACGGTGCTGTGTAATCCGCACAAAATTTTACA   657
AH007417.2                 ------------------------------------------------------------   343
AH007416.2                 ------------------------------------------------------------   343
AH007415.2                 ------------------------------------------------------------   343
AH007265.2                 ------------------------------------------------------------   343
AH007264.2                 ------------------------------------------------------------   343
AH007263.2                 ------------------------------------------------------------   343
AH007228.2                 ------------------------------------------------------------   343
AH007227.2                 ------------------------------------------------------------   343
AH007223.2                 ------------------------------------------------------------   343
AH007222.2                 ------------------------------------------------------------   319
AH007221.2                 ------------------------------------------------------------   346
AH007220.2                 ------------------------------------------------------------   346
AH007219.2                 ------------------------------------------------------------   343
AH007218.2                 ------------------------------------------------------------   343
AH007217.2                 ------------------------------------------------------------   343

Consensus                  ------------------------------------------------------------   364
ENA|AF059535|AF059535.1 TTTCTTTTTGGGGTGTTATTTGAAGGGGAAAGGTCTGACGTGGAAAACCCTCATGCATGG   717
AH007417.2                 ------------------------------------------------------------   343
AH007416.2                 ------------------------------------------------------------   343
AH007415.2                 ------------------------------------------------------------   343
AH007265.2                 ------------------------------------------------------------   343
AH007264.2                 ------------------------------------------------------------   343
AH007263.2                 ------------------------------------------------------------   343
AH007228.2                 ------------------------------------------------------------   343
AH007227.2                 ------------------------------------------------------------   343
AH007223.2                 ------------------------------------------------------------   343
AH007222.2                 ------------------------------------------------------------   319
AH007221.2                 ------------------------------------------------------------   346
AH007220.2                 ------------------------------------------------------------   346
AH007219.2                 ------------------------------------------------------------   343
AH007218.2                 ------------------------------------------------------------   343
AH007217.2                 ------------------------------------------------------------   343

Consensus                  -------G----------------------------------------------------   365
ENA|AF059535|AF059535.1 GGGCTTTGTCATATCAATACAACTACTGTGACTGAGAATGACTTCTGTACCTCCTCCGAC   777
AH007417.2                 -------N----------------------------------------------------   344
AH007416.2                 -------N----------------------------------------------------   344
AH007415.2                 -------N----------------------------------------------------   344
AH007265.2                 -------N----------------------------------------------------   344
AH007264.2                 -------N----------------------------------------------------   344
AH007263.2                 -------N----------------------------------------------------   344
AH007228.2                 -------N----------------------------------------------------   344
AH007227.2                 -------N----------------------------------------------------   344
AH007223.2                 -------N----------------------------------------------------   344
AH007222.2                 -------N----------------------------------------------------   320
AH007221.2                 -------N----------------------------------------------------   347
AH007220.2                 -------N----------------------------------------------------   347
AH007219.2                 -------N----------------------------------------------------   344
AH007218.2                 -------N----------------------------------------------------   344
AH007217.2                 -------N----------------------------------------------------   344

Consensus                  -------------------------------------G----------------------   366
ENA|AF059535|AF059535.1 TGGCCTTGCGCTGCCGGCAAAAAATACAGCCCTCGAGGACCCATCCAGCTCACCCAGTCA   837
```

```
AH007417.2                -------------------------------------N----------------------   345
AH007416.2                -------------------------------------N----------------------   345
AH007415.2                -------------------------------------N----------------------   345
AH007265.2                -------------------------------------N----------------------   345
AH007264.2                -------------------------------------N----------------------   345
AH007263.2                -------------------------------------N----------------------   345
AH007228.2                -------------------------------------N----------------------   345
AH007227.2                -------------------------------------N----------------------   345
AH007223.2                -------------------------------------N----------------------   345
AH007222.2                -------------------------------------N----------------------   321
AH007221.2                -------------------------------------N----------------------   348
AH007220.2                -------------------------------------N----------------------   348
AH007219.2                -------------------------------------N----------------------   345
AH007218.2                -------------------------------------N----------------------   345
AH007217.2                -------------------------------------N----------------------   345

Consensus                 ----------------------T----------------------G--------------   368
ENA|AF059535|AF059535.1   GTGCATAGTAGTTTTGTTCTTATATTTATTCATTCTAAAGTAGCAGTCAAATAATATTAT   897
AH007417.2                ----------------------N----------------------N--------------   347
AH007416.2                ----------------------N----------------------N--------------   347
AH007415.2                ----------------------N----------------------N--------------   347
AH007265.2                ----------------------N----------------------N--------------   347
AH007264.2                ----------------------N----------------------N--------------   347
AH007263.2                ----------------------N----------------------N--------------   347
AH007228.2                ----------------------N----------------------N--------------   347
AH007227.2                ----------------------N----------------------N--------------   347
AH007223.2                ----------------------N----------------------N--------------   347
AH007222.2                ----------------------N-------------------------------------   322
AH007221.2                ----------------------N----------------------N----------N---   351
AH007220.2                ----------------------N----------------------N----------N---   351
AH007219.2                ----------------------N----------------------N----------N---   348
AH007218.2                ----------------------N----------------------N----------N---   348
AH007217.2                ----------------------N----------------------N----------N---   348

Consensus                 ---TTGTTTTTTTTTATAGCAACTTCAACTACGGACTTGCCGGCCAAGCCATTGGAGAGG   425
ENA|AF059535|AF059535.1   TTGTTGTTTTTTTTTATAGCAACTTCAACTACGGACTTGCCGGCCAAGCCATTGGAGAGG   957
AH007417.2                --NNNNNNNNNNNNNNNNNNNNNNNNNNNNNNNNNNNNNNNNNNNNNNNNNNNNNNNNNN   405
AH007416.2                --NNNNNNNNNNNNNNNNNNNNNNNNNNNNNNNNNNNNNNNNNNNNNNNNNNNNNNNNNN   405
AH007415.2                --NNNNNNNNNNNNNNNNNNNNNNNNNNNNNNNNNNNNNNNNNNNNNNNNNNNNNNNNNN   405
AH007265.2                --NNNNNNNNNNNNNNNNNNNNNNNNNNNNNNNNNNNNNNNNNNNNNNNNNNNNNNNNNN   405
AH007264.2                --NNNNNNNNNNNNNNNNNNNNNNNNNNNNNNNNNNNNNNNNNNNNNNNNNNNNNNNNNN   405
AH007263.2                --NNNNNNNNNNNNNNNNNNNNNNNNNNNNNNNNNNNNNNNNNNNNNNNNNNNNNNNNNN   405
AH007228.2                --NNNNNNNNNNNNNNNNNNNNNNNNNNNNNNNNNNNNNNNNNNNNNNNNNNNNNNNNNN   405
AH007227.2                --NNNNNNNNNNNNNNNNNNNNNNNNNNNNNNNNNNNNNNNNNNNNNNNNNNNNNNNNNN   405
AH007223.2                --NNNNNNNNNNNNNNNNNNNNNNNNNNNNNNNNNNNNNNNNNNNNNNNNNNNNNNNNNN   405
AH007222.2                ----NNNNNNNNNNNNNNNNNNNNNNNNNNNNNNNNNNNNNNNNNNNNNNNNNNNNNNNN   378
AH007221.2                ---NNNNNNNNNNNNNNNNNNNNNNNNNNNNNNNNNNNNNNNNNNNNNNNNNNNNNNNNN   408
AH007220.2                ---NNNNNNNNNNNNNNNNNNNNNNNNNNNNNNNNNNNNNNNNNNNNNNNNNNNNNNNNN   408
AH007219.2                ---NNNNNNNNNNNNNNNNNNNNNNNNNNNNNNNNNNNNNNNNNNNNNNNNNNNNNNNNN   405
AH007218.2                ---NNNNNNNNNNNNNNNNNNNNNNNNNNNNNNNNNNNNNNNNNNNNNNNNNNNNNNNNN   405
AH007217.2                ---NNNNNNNNNNNNNNNNNNNNNNNNNNNNNNNNNNNNNNNNNNNNNNNNNNNNNNNNN   405

Consensus                 ACCTGATTCAGAACCCTGACTTGGTAGAAAAGGATCCAATCATATCATTCAAGACGGCCT   485
ENA|AF059535|AF059535.1   ACCTGATTCAGAACCCTGACTTGGTAGAAAAGGATCCAATCATATCATTCAAGACGGCCT  1017
AH007417.2                NNNNNNNNNNNNNNNNNNNNNNNNNNNNNNNGGATCCAATCATATCATTCAAGACGGCCT   465
AH007416.2                NNNNNNNNNNNNNNNNNNNNNNNNNNNNNNNGGACCCAATCATATCATTCAAGACGGCCT   465
AH007415.2                NNNNNNNNNNNNNNNNNNNNNNNNNNNNNNNGGACCCAATCATATCATTCAAGACGGCCT   465
AH007265.2                NNNNNNNNNNNNNNNNNNNNNNNNNNNNNNNGGACCCAATCATATCATTCAAGACGGCCT   465
AH007264.2                NNNNNNNNNNNNNNNNNNNNNNNNNNNNNNNGGATCCAATCATATCATTCAAGACGGCCT   465
AH007263.2                NNNNNNNNNNNNNNNNNNNNNNNNNNNNNNNGGACCCAATCATATCATTCAAGACGGCCT   465
AH007228.2                NNNNNNNNNNNNNNNNNNNNNNNNNNNNNNNGGATCCAATCATATCATTCAAGACGGCCT   465
AH007227.2                NNNNNNNNNNNNNNNNNNNNNNNNNNNNNNNGGATCCAATCATATCATTCAAGACGGCCT   465
AH007223.2                NNNNNNNNNNNNNNNNNNNNNNNNNNNNNNNGGATCCAATCATATCATTCAAGACGGCCT   465
AH007222.2                NNNNNNNNNNNNNNNNNNNNNNNNNNNNNNNGGATCCAATCATATCATTCAAGACGGCCT   438
AH007221.2                NNNNNNNNNNNNNNNNNNNNNNNNNNNNNNNGGATCCAATCATATCATTCAAGACGGCCT   468
AH007220.2                NNNNNNNNNNNNNNNNNNNNNNNNNNNNNNNGGATCCAATCATATCATTCAAGACGGCCT   468
AH007219.2                NNNNNNNNNNNNNNNNNNNNNNNNNNNNNNNGGATCCAATCATATCATTCAAGACGGCCT   465
AH007218.2                NNNNNNNNNNNNNNNNNNNNNNNNNNNNNNNGGATCCAATCATATCATTCAAGACGGCCT   465
AH007217.2                NNNNNNNNNNNNNNNNNNNNNNNNNNNNNNNGGATCCAATCATATCATTCAAGACGGCCT   465

Consensus                 TGTGGTTCTGGATGTCCCAGCACGACAACAAACCTTCATGCCATGACATTGTCCTCAATG   545
ENA|AF059535|AF059535.1   TGTGGTTCTGGATGTCCCAGCACGACAACAAACCTTCATGCCATGACATTGTCCTCAATG  1077
AH007417.2                TGTGGTTCTGGATGTCCCAGCACGACAACAAACCTTCATGCCATGACATTGTCCTCAATG   525
AH007416.2                TGTGGTTCTGGATGTCCCAGCACGACAACAAACCTTCATGCCATGACATTGTCCTCAATG   525
AH007415.2                TGTGGTTCTGGATGTCCCAGCACGACAACAAACCTTCATGCCATGACATTGTCCTCAATG   525
AH007265.2                TGTGGTTCTGGATGTCCCAGCACGACAACAAACCTTCATGCCATGACATTGTCCTCAATG   525
AH007264.2                TGTGGTTCTGGATGTCCCAGCACGACAACAAACCTTCATGCCATGACATTGTCCTCAATG   525
AH007263.2                TGTGGTTCTGGATGTCCCAGCACGACAACAAACCTTCATGCCATGACATTGTCCTCAATG   525
AH007228.2                TGTGGTTCTGGATGTCCCAGCACGACAACAAACCTTCATGCCATGACATTGTCCTCAATG   525
AH007227.2                TGTGGTTCTGGATGTCCCAGCACGACAACAAACCTTCATGCCATGACATTGTCCTCAATG   525
AH007223.2                TGTGGTTCTGGATATCCCAGCACGACAACAAACCTTCATGCCATGACATTGTCCTCAATG   525
```

```
AH007222.2                 TGTGGTTCTGGATGTCCCAGCACGACAACAAACCTTCATGCCATGACATTGTCCTCAATG   498
AH007221.2                 TGTGGTTCTGGATGTCCCAGCACGACAACAAACCTTCATGCCATGACATTGTCCTCAATG   528
AH007220.2                 TGTGGTTCTGGATGTCCCAGCACGACAACAAACCTTCATGCCATGACATTGTCCTCAATG   528
AH007219.2                 TGTGGTTCTGGATGTCCCAGCACGACAACAAACCTTCATGCCATGACATTGTCCTCAATG   525
AH007218.2                 TGTGGTTCTGGATGTCCCAGCACGACAACAAACCTTCATGCCATGACATTGTCCTCAATG   525
AH007217.2                 TGTGGTTCTGGATGTCCCAGCACGACAACAAACCTTCATGCCATGACATTGTCCTCAATG   525

Consensus                  CCAACTCCGCCGCGAACAGAATCCCAAACAAAGGTGTGATCGGCAACATTATTAGCCGCG   605
ENA|AF059535|AF059535.1 CCAACTCCGCCGCGAACAGAATCCCAAACAAAGGTGTGATCGGCAACATTATTAGCCGCG  1137
AH007417.2                 CCAACTCCGCCGCGAACAGAATCCCAAACAAAGGTGTGATCGGCAGCATTATTAGCCGCG   585
AH007416.2                 CCAACTCCGCCGCTAACACAGCCCCAAACAAAGGTGTGATCAGCAACATTATTAGCCGCG   585
AH007415.2                 CCAACTCCGCCGCTAACACAGCCCCAAACAAAGGTGTGATCAGCAACATTATTAGCCGCG   585
AH007265.2                 CCAACTCCGCCGCTAACACAGCCCCAAACAAAGGTGTGATCAGCAACATTATTAGCCGCG   585
AH007264.2                 CCAACTCCGCCGCGAACAGAATCCCAAACAAAGGTGTGATCGGCAGCATTATTAGCCGCG   585
AH007263.2                 CCAACTCCGCCGCTAACACAGCCCCAAACAAAGGTGTGATCAGCAACATTATTAGCCGCG   585
AH007228.2                 CCAACTCCGCCGCGAACAGAATCCCAAACAAAGGTGTGATCGGCAACATTATTAGCCGCG   585
AH007227.2                 CCAACTCCGCCGCGAACAGAATCCCAAACAAAGGTGTGATCGGCAACATTATTAGCCGCG   585
AH007223.2                 CCAACTCCGCCGCGAACAGAATCCCAAACAAAGGTGTGATCGGCAACATTATTAGCCGCG   585
AH007222.2                 CCAACTCCGCCGCTAACACAGCCCCAAACAAAGGTGTGATCAGCAACATTATTAGCCGCG   558
AH007221.2                 CCAACTCCGCCGCTAACACAGCCCCAAACAAAGGTGTGATCGGCAACATTATTAGCCGCG   588
AH007220.2                 CCAACTCCGCCGCTAACACAGCCCCAAACAAAGGTGTGATCGGCAACATTATTAGCCGCG   588
AH007219.2                 CCAACTCCGCCGCGAACAGAATCCCAAACAAAGGTGTGATCGGCAGCATTATTAGCCGCG   585
AH007218.2                 CCAACTCCGCCGCGAACAGAATCCCAAACAAAGGTGTGATCGGCAGCATTATTAGCCGCG   585
AH007217.2                 CCAACTCCGCCGCGAACAGAATCCCAAACAAAGGTGTGATCGGCAACATTATTAGCCGCG   585

Consensus                  CTTTTGGGCACGACGACTTTGCCGTTAGATCTTCAAGCATCGGATTTTACAAGAGGTACT   665
ENA|AF059535|AF059535.1 CTTTTGGGCACGACGACTTTGCCGTTAGATCTTCAAGCATCGGATTTTACAAGAGGTACT  1197
AH007417.2                 CTTTTGGGCACGACGACTTTGCCGTTAGATCTTCAAGCATCGGATTTTACAAGAGGTACT   645
AH007416.2                 CTTTTGGGCACGACGACTTTGCCGTTAGGTCTTCAAGCATCGGATTTTACAAGAGGTACT   645
AH007415.2                 CTTTTGGGCACGACGACTTTGCCGTTAGGTCTTCAAGCATCGGATTTTACAAGAGGTACT   645
AH007265.2                 CTTTTGGGCACGACGACTTTGACGTTAGATCTTCAAGCATCGGATTTTACAAGAGGTACT   645
AH007264.2                 CTTTTGGGCACGACGACTTTGCCGTTAGATCTTCAAGCATCGGATTTTACAAGAGGTACT   645
AH007263.2                 CTTTTGGGCACGACGACTTTGACGTTAGATCTTCAAGCATCGGATTTTACAAGAGGTACT   645
AH007228.2                 CTTTTGGGCACGACGACTTTGCCGTTAGATCTTCAAGCATCGGATTTTACAAGAGGTACT   645
AH007227.2                 CTTTTGGGCACGACGACTTTGCCGTTAGATCTTCAAGCATCGGATTTTACAAGAGGTACT   645
AH007223.2                 CTTTTGGGCACGACGACTTTGCCGTTAGATCTTCAAGCATCGGATTTTACAAGAGGTACT   645
AH007222.2                 CTTTTGGGCACGACGACTTTGCCGTTAGATCTTCAAGCATCGGATTTTACAAGAGGTACT   618
AH007221.2                 CTTTTGGGCACGACGACTTTGCCGTTAGATCTTCAAGCATCGGATTTTACAAGAGGTACT   648
AH007220.2                 CTTTTGGGCACGACGACTTTGCCGTTAGATCTTCAAGCATCGGATTTTACAAGAGGTACT   648
AH007219.2                 CTTTTGGGCACGACGACTTTGCCGTTAGATCTTCAAGCATCGGATTTTACAAGAGGTACT   645
AH007218.2                 CTTTTGGGCACGACGACTTTGCCGTTAGATCTTCAAGCATCGGATTTTACAAGAGGTACT   645
AH007217.2                 CTTTTGGGCACGACGACTTTGCCGTTAGATCTTCAAGCATCGGATTTTACAAGAGGTACT   645

Consensus                  GCGACATGCTGGGAGTGAGCTATGGACATGACTTGAAGTACTGGTTCGATAACACTCCAT   725
ENA|AF059535|AF059535.1 GCGACATGCTGGGAGTGAGCTATGGACATGACTTGAAGTACTGGTTCGATAACACTCCAT  1257
AH007417.2                 GCGACATGCTGGGAGTGAGCTATGGACATGACTTGAAGTACTGGTTCGATAACACTCCAT   705
AH007416.2                 GCGACATGCTGGGAGTGAGCTATGGACATGACTTGAAGTACTGGTTCGATAACACTCCAT   705
AH007415.2                 GCGACATGCTGGGAGTGAGCTATGGACATGACTTGAAGTACTGGTTCGATAACACTCCAT   705
AH007265.2                 GCGACATGCTGGGAGTGAGCTATGGACATGACTTGAAGTACTGGTTCGATAACACTCCAT   705
AH007264.2                 GCGACATGCTGGGAGTGAGCTATGGACATGACTTGAAGTACTGGTTCGATAACACTCCAT   705
AH007263.2                 GCGACATGCTGGGAGTGAGCTATGGACATGACTTGAAGTACTGGTTCGATAACACTCCAT   705
AH007228.2                 GCGACATGCTGGGAGTGAGCTATGGACATGACTTGAAGTACTGGTTCGATAACACTCCAT   705
AH007227.2                 GCGACATGCTGGGAGTGAGCTATGGACATGACTTGAAGTACTGGTTCGATAACACTCCAT   705
AH007223.2                 GCGACATGCTGGGAATGAGCTATGGACATGACTTGAAGTACTGGTTCGATAACACTCCAT   705
AH007222.2                 GCGACATGCTGGGAGTTAGCTATGGACATGACTTGAAGTACTGGTTCGATAACACTCCAT   678
AH007221.2                 GCGACATGCTGGGAGTGAGCTATGGACATGACTTGAAGTACTGGTTCGATAACACTCCAT   708
AH007220.2                 GCGACATGCTGGGAGTGAGCTATGGACATGACTTGAAGTACTGGTTCGATAACACTCCAT   708
AH007219.2                 GCGACATGCTGGGAGTGAGCTATGGACATGACTTGAAGTACTGGTTCGATAACACTCCAT   705
AH007218.2                 GCGACATGCTGGGAGTGAGCTATGGACATGACTTGAAGTACTGGTTCGATAACACTCCAT   705
AH007217.2                 GCGACATGCTGGGAGTGAGCTATGGACATGACTTGAAGTACTGGTTCGATAACACTCCAT   705

Consensus                  CATCGGAGTTCCAACGCATCCAAATGCGTGTTGCGGCGTA-AAACAAGCTAGTCCTCCCC   784
ENA|AF059535|AF059535.1 CATCGGAGTTCCAACGCATCCAAATGCGTGTTGCGGCGTA-AAACAAGCTAGTCCTCCCC  1316
AH007417.2                 CATCGGAGTTCCAACGCATCCAAATGCGTGTTGCGGCGTA-AAACAAGCTAGTCCTCCCC   764
AH007416.2                 CATCGGAGTTCCAACGCATCCAAATGCGTGTTGCGGCGTAAAAACAAGCTAGTCCTCCCC   765
AH007415.2                 CATCGGAGTTCCAACGCATCCAAATGCGTGTTGCGGCGTAAAAACAAGCTAGTCCTCCCC   765
AH007265.2                 CATCGGAGTTCCAACGCATCCAAATGCGTGTTGCGGCGTA-AAACAAGCTAGTCCTCCCC   764
AH007264.2                 CATCGGAGTTCCAACGCATCCAAATGCGTGTTGCGGCGTA-AAACAAGCTAGTCCTCCCC   764
AH007263.2                 CATCGGAGTTCCAACGCATCCAAATGCGTGTTGCGGCGTAAAAACAAGCTAGTCCTCCCC   765
AH007228.2                 CATCGGAGTTCCAACGCATCCAAATGCGTGTTGCGGCGTA-AAACAAGCTAGTCCTCCCC   764
AH007227.2                 CATCGGAGTTCCAACGCATCCAAATGCGTGTTGCGGCGTA-AAACAAGCTAGTCCTCCCC   764
AH007223.2                 CATCGGAGTTCCAACGCATCCAAATGCGTGTTGCGGCGTA-AAACAAGCTAGTCCTCCCC   764
AH007222.2                 CATCAGAGTTACAACGCATCCAAATGCGTGTGGCGGCGTAAAAACAAGCTAGTCCTCCCC   738
AH007221.2                 CATCGGAGTTCCAACGCATCCAAATGCGTGTTGCGGCGTA-AAACAAGCTAGTCCTCCCC   767
AH007220.2                 CATCGGAGTTCCAACGCATCCAAATGCGTGTTGCGGCGTA-AAACAAGCTAGTCCTCCCC   767
AH007219.2                 CATCGGAGTTCCAACGCATCCAAATGCGTGTTGCGGCGTA-AAACAAGCTAGTCCTCCCC   764
AH007218.2                 CATCGGAGTTCCAACGCATCCAAATGCGTGTTGCGGCGTA-AAACAAGCTAGTCCTCCCC   764
AH007217.2                 CATCGGAGTTCCAACGCATCCAAATGCGTGTTGCGGCGTA-AAACAAGCTAGTCCTCCCC   764

Consensus                  AAGTGGCTCTCTAGTAGTAAGAGTAGCTCTCTCATAGCGAGAGAGCGGCATGTTGAATCC   844
ENA|AF059535|AF059535.1 AAGTGGCTCTCTAGTAGTAAGAGTAGCTCTCTCATAGCTAGAGAGCGGCATGTTGAATCC  1376
```

```
AH007417.2                 AAGTGGCTCTCTAGTAGTAAGAGTAGCTCTCTCATAGCGAGAGAGCGGCATGTTGAATCC   824
AH007416.2                 AAGTGGCTGTCTTGTTGTAAGAGTAGCTCTCTCATAGCGAGAGAGCGGCATGTTGAATCC   825
AH007415.2                 AAGTGGCTCTCTAGTAGTAAGAGTAGCTCTCTCATAGCGAGAGAGCGGCATGTTGAATCC   825
AH007265.2                 AAGTGGCTCTCTAGTAGTAAGAGTAGCTCTCTCATAGCGAGAGAGCGGCATGTTGAATCC   824
AH007264.2                 AAGTGGCTCTCTAGTAGTAAGAGTAGCTCTCTCATAGCGAGAGAGCGGCATGTTGAATCC   824
AH007263.2                 AAGTGGCTCTCTAGTAGTAAGAGTAGCTCTCTCATAGCGAGAGAGCGGCATGTTGAATCC   825
AH007228.2                 AAGTGGCTCTCTAGTAGTAAGAGTAGCTCTCTCATAGCTAGAGAGCGGCATGTTGAATCC   824
AH007227.2                 AAGTGGCTCTCTAGTAGTAAGAGTAGCTCTCTCATAGCGAGAGAGCGGCATGTTGAATCC   824
AH007223.2                 AAGTCGCTCTCTAGTAGTAAGAGTA----TGT--TA-C---------GCATGTTGAATCC   808
AH007222.2                 AAGTGGCTGTCTTGTTGTAAGAGTAGCTCTCTCATAGCGAGAGAGCGGCATGTTGAATCC   798
AH007221.2                 AAGTGGCTCTCTAGTAGTAAGAGTAGCTCTCTCATAGCGAGAGAGCGGCATGTTGAATCC   827
AH007220.2                 AAGTGGCTCTCTAGTAGTAAGAGTAGCTCTCTCATAGCGAGAGAGCGGCATGTTGAATCC   827
AH007219.2                 AAGTGGCTCTCTAGTAGTAAGAGTAGCTCTCTCATAGCGAGAGAGCGGCATGTTGAATCC   824
AH007218.2                 AAGTGGCTCTCTAGTAGTAAGAGTAGCTCTCTCATAGCTAGAGAGCGGCATGTTGAATCC   824
AH007217.2                 AAGTCGCTCTCTAGTAGTAAGAGTA----TGT--TA-C---------GCATGTTGAATCC   808

Consensus                  CTGTTATGCTATGTAATATTATGTTACGCATGTATGTTAGAAACATATATGTGTGATTTT   904
ENA|AF059535|AF059535.1 CTGTTATGCTATGTAATATTATGTTACGCATGTATGTTAGAAACATATATGTGTGATTTT  1436
AH007417.2                 CTGTTATGCTATGTAATATTATGTTACGCATGTATGTTAGAAACATATATGTGTGATTTT   884
AH007416.2                 ATGTTATGCTATGTAATATTATGTTACGCATGTATGTTAGAAACATATATGTGTGATTTT   885
AH007415.2                 ATGTTATGCTATGTAATATTATGTTACGCATGTATGTTAGAAACATATATGTGTGATTTT   885
AH007265.2                 CTGTTATGCTATGTAATATTATGTTACGCATGTATGTTAGAAACATATATGTGTGATTTT   884
AH007264.2                 CTGTTATGCTATGTAATATTATGTTACGCATGTATGTTAGAAACATATATGTGTGATTTT   884
AH007263.2                 ATGTTATGCTATGTAATATTATGTTACGCATGTATGTTAGAAACATATATGTGTGATTTT   885
AH007228.2                 CTGTTATGCTATGTAATATTATGTTACGCATGTATGTTAGAAACATATATGTGTGATTTT   884
AH007227.2                 CTGTTATGCTATGTAATATTATGTTACGCATGTATGTTAGAAACATATATGTGTGATTTT   884
AH007223.2                 ATGTTATGCTATGTAATATTATGTTACGCATGTATGTTAGAAACATTTATGTGTGATTTT   868
AH007222.2                 ATGTTATGCTATGTAATATTATGTTACGCATGTATGTTAGAAACATATATGTGTGATTTT   858
AH007221.2                 ATGTTATGCTATGTAATATTATGTTACGCATGTATGTTAGAAACATATATGTGTGATTTT   887
AH007220.2                 CTGTTATGCTATGTAATATTATGTTACGCATGTATGTTAGAAACATATATGTGTGATTTT   887
AH007219.2                 CTGTTATGCTATGTAATATTATGTTACGCATGTATGTTAGAAACATATATGTGTGATTTT   884
AH007218.2                 CTGTTATGCTATGTAATATTATGTTACGCATGTATGTTAGAAACATATATGTGTGATTTT   884
AH007217.2                 ATGTTATGCTATGTAATATTATGTTACGCATGTATGTTAGAAACATTTATGTGTGATTTT   868

Consensus                  CTAGCTCTTACGAG                                                 918
ENA|AF059535|AF059535.1 CTAGCTCTTACGAG                                                1450
AH007417.2                 CTAGCTCTTACGAG                                                 898
AH007416.2                 CTAGCTCTTACGAG                                                 899
AH007415.2                 CTAGCTCTTACGAG                                                 899
AH007265.2                 CTAGCTCTTACGAG                                                 898
AH007264.2                 CTAGCTCTTACGAG                                                 898
AH007263.2                 CTAGCTCTTACGAG                                                 899
AH007228.2                 CTAGCTCTTACGAG                                                 898
AH007227.2                 CTAGCTCTTACGAG                                                 898
AH007223.2                 CTAGCTCTTACGAG                                                 882
AH007222.2                 CTAGCTCTTACGAG                                                 872
AH007221.2                 CTAGCTCTTACGAG                                                 901
AH007220.2                 CTAGCTCTTACGAG                                                 901
AH007219.2                 CTAGCTCTTACGAG                                                 898
AH007218.2                 CTAGCTCTTACGAG                                                 898
AH007217.2                 CTAGCTCTTACGAG                                                 882
```

Figure S3 Location of UDA-sequences from Does et al 1999 (N-ambiguities removed) in the new nettle genome assembly [50].
(Hirabayashi et al 2025 https://doi.org/10.3390/plants14010124)
Search done with a local blast system using Geneious Prime® 2025.0.3 software

Chromosome matched UDA-accession	Location minimum	Location maximum	Length of searched sequence
Urtica_dioica_female_chr_11-7263.2	8,349,349	8,349,811	463
Urtica_dioica_female_chr_11-7265.2	8,349,349	8,349,811	463
Urtica_dioica_female_chr_11-7415.2	8,349,349	8,349,811	463
Urtica_dioica_female_chr_11-7416.2	8,349,349	8,349,811	463
Urtica_dioica_female_chr_11-7217.2	8,448,536	8,448,981	446
Urtica_dioica_female_chr_11-7223.2	8,448,536	8,448,981	446
Urtica_dioica_female_chr_11-59535	8,490,116	8,491,569	1,454
Urtica_dioica_female_chr_11-7218.2	8,491,108	8,491,569	462
Urtica_dioica_female_chr_11-7219.2	8,491,108	8,491,569	462
Urtica_dioica_female_chr_11-7220.2	8,491,108	8,491,569	462
Urtica_dioica_female_chr_11-7221.2	8,491,108	8,491,569	462
Urtica_dioica_female_chr_11-7222.2	8,491,108	8,491,569	462
Urtica_dioica_female_chr_11-7227.2	8,491,108	8,491,569	462
Urtica_dioica_female_chr_11-7228.2	8,491,108	8,491,569	462
Urtica_dioica_female_chr_11-7264.2	8,491,108	8,491,569	462
Urtica_dioica_female_chr_11-7417.2	8,491,108	8,491,569	462
